# Preventive Effects of Kaempferol on High-Fat Diet-Induced Obesity Complications in C57BL/6 Mice

**DOI:** 10.1155/2020/4532482

**Published:** 2020-04-07

**Authors:** Tieqiao Wang, Qiaomin Wu, Tingqi Zhao

**Affiliations:** ^1^The Affiliated Hospital of Medical School, Ningbo University, Ningbo, Zhejiang, China; ^2^Endocrinology Department, First Affiliated Hospital of Zhejiang Chinese Medical University, Hangzhou, Zhejiang, China

## Abstract

Kaempferol is a dietary flavanol that regulates cellular lipid and glucose metabolism. Its mechanism of action in preventing hepatic steatosis and obesity-related disorders has yet to be clarified. The purpose of this research was to examine kaempferol's antiobesity effects in high-fat diet- (HFD-) fed mice and to investigate its impact on their gut microbiota. Using a completely randomized design, 30 mice were equally assigned to a control group, receiving a low-fat diet, an HFD group, receiving a high-fat diet, and an HFD+kaempferol group, receiving a high-fat diet and kaempferol doses of 200 mg/kg in the diet. After eight weeks, the HFD mice displayed substantial body and liver weight gain and high blood glucose and serum cholesterol levels. However, treatment with kaempferol moderated body and liver weight gain and elevation of blood glucose and serum cholesterol and triglyceride levels. Examination of 16S ribosomal RNA showed that HFD mice exhibited decreased microbial diversity, but kaempferol treatment maintained it to nearly the same levels as those in the control group. In conclusion, kaempferol can protect against obesity and insulin resistance in mice on a high-fat diet, partly through regulating their gut microbiota and moderating the decrease in insulin resistance.

## 1. Introduction

Obesity is the result of the accumulation of excessive amounts of fat in adipose tissue. Obesity is linked to many risk factors, including cardiovascular diseases, hypertension, hepatic steatosis, insulin resistance, and dyslipidemia [1, 2]. The prevalence of obesity and associated metabolic disorders (e.g., type 2 diabetes mellitus and cardiovascular diseases) has increased considerably in the last century, not only in developed but also in developing countries [3]. Rat studies indicate that long-term intake of a high-fat diet (HFD) can lead to impaired lipid metabolism, as indicated by elevated total cholesterol (TC), triglyceride (TG), and low-density lipoprotein (LDL) levels [4]. In addition to the disruption of lipid metabolism, low-grade chronic inflammation has been identified as a potential mechanism of obesity-related conditions [5].

It has been hypothesized that excessive accumulation of adipose tissue is a precursor to the production of proinflammatory cytokines, which can lead to hypertension and insulin resistance [6]. Moreover, data from both human and animal studies suggest a close connection between the emergence of an obese phenotype and the gut microbiome. The microbiota of obese mice is marked by an increase in the relative abundance of Firmicutes and a decrease in Bacteroidetes [7]. The literature suggests that substantial changes occur in the composition of the gut microbiota within 24 h of a switch to an HFD [8]. Presently, various medications are administered to prevent, treat, and manage obesity. However, pharmacological agents of this kind are associated with adverse side effects and are not always effective [9]. In view of this, many scholars have focused their efforts on developing novel therapeutic agents for obesity-related complications.

Polyphenols have attracted considerable interest in recent years owing to their potential use in the management of diabetes [10, 11]. As secondary plant metabolites, polyphenolic compounds are the principal source of dietary antioxidants for humans, in whom the normal intake of polyphenols is approximately 1 g per day [12]. Noteworthily, robust research findings indicate that dietary polyphenols mitigate HFD-induced obesity by regulating the gut microbiota [13, 14].

Kaempferol (3,5,7-trihydroxy-2-(4-hydroxyphenyl)-4H-1-benzopyran-4-one) is a flavanol that has been identified in a variety of edible plants and traditional medicines. Studies suggest that it may be an effective agent against obesity [15, 16]. In a study involving HFD obese mice, the administration of kaempferol in daily doses of 50 mg/kg was associated with significant improvements in blood glucose control, which were in turn attributed to reduced hepatic glucose production and enhanced whole-body insulin sensitivity [16]. Significantly, no alterations were observed in terms of body weight gain, adiposity, or food consumption. However, the mechanism of action that underpins the antiobesity activity of this flavanol is not yet understood.

With the above considerations in mind, the purpose of this study was to identify notable metabolites and to investigate the potential preventive mechanism of action associated with kaempferol in HFD-induced obesity in C57BL/6 mice.

## 2. Materials and Methods

### 2.1. Animal Care and Experimental Design

Approval was obtained from Zhejiang Traditional Chinese Hospital's Animal Use and Welfare Committee. Thirty C57BL/6 mice aged nine weeks were procured from Nanjing University's Model Animal Research Center, stored in standard, pathogen-free conditions (temperature: 22 ± 2°C; relative humidity: 50 ± 5%; and 12 h light/dark cycles), and provided with water and food *ad libitum*.

Using a completely randomized design, each mouse was assigned to one of the following three groups (*n* = 10 each): a control group, receiving a low-fat diet; an HFD group, receiving a high-fat diet; and an HFD+kaempferol group, receiving a high-fat diet and 200 mg/kg kaempferol in the diet. The low-fat diet consisted of 10% (kcal%) fat, 20% protein, and 70% carbohydrate, whereas the HFD consisted of 45% fat, 20% protein, and 35% carbohydrate (Research Diets, Inc., New Brunswick, NJ, USA). The experimental period was eight weeks.

Upon completion of the experiment, the mice were subjected to fasting for 4 h, after which blood samples were taken from the retro-orbital sinus. After cervical dislocation, the inguinal white adipose tissue (iWAT), perirenal white adipose tissue (pWAT), epididymal white adipose tissue (eWAT), and finally the liver were weighed.

### 2.2. Biochemical Analysis

The blood samples were centrifuged at 3,000 rpm at 4°C for 10 min to obtain serum. A Beckman CX4 biochemical analyzer (Beckman Coulter, Brea, CA, USA) and kits manufactured by Roche (Basel, Switzerland) were used to measure serum triglyceride (TG), glucose, high-density lipoprotein (HDL), low-density lipoprotein (LDL), and total cholesterol (TC) levels.

### 2.3. Glucose and Insulin Tolerance Measurements

A Glucometer Elite (Bayer, Leverkusen, Germany) was used to measure blood glucose levels in blood obtained from the tail vein. For the glucose tolerance tests (GTT), blood glucose was measured 0, 15, 30, 60, and 120 min after administration of bolus intraperitoneal glucose in a dose of 2 g/kg to mice subjected to overnight fasting (12 h). For the insulin tolerance tests (ITT), blood glucose was measured 0, 15, 30, 60, and 90 min after administration of an intraperitoneal injection of regular human insulin in a dose of 0.80 U/kg to mice subjected to fasting for 6 h. The homeostasis model assessment of the insulin resistance index was calculated according to the following standard formula: basal glucose (mM) and basal insulin (mU/L)/22.5.

### 2.4. Gut Microbiota Analysis

A QIAamp DNA Stool Mini Kit (Qiagen, Duesseldorf, Germany) was used to extract total DNA from fecal samples according to the manufacturer's instructions (QIAamp DNA Stool Handbook, 04/2010). A MiSeq instrument (Illumina Inc., San Diego, CA, USA) was used by Majorbio (Shanghai, China) to perform 16S ribosomal RNA (rRNA) high-throughput sequencing. Amplification of the variable regions V3-V4 of the 16S rRNA genes was performed with barcode-indexed primers 338F and 806R. The forward and reverse primer sequences were 5′-ACTCCTACGGGAGGCAGCA-3′ and 5′-GGACTACHVGGGTWTCTAAT-3′, respectively. A 2% gel extraction kit (AxyPrep DNA Gel Extraction Kit; Axygen Biosciences, Union City, CA, USA) was used to purify the amplicons according to the manufacturer's instructions. Polymerase chain reaction (PCR) product concentrations were assessed with a QuantiFluor™-ST (Promega, Madison, WI, USA), and product normalization was performed at equimolar concentrations with paired-end sequencing (2 × 250) using the MiSeq platform (Illumina Inc.) according to standard protocols. After sequencing, raw data splicing and filtration were performed to obtain cleaned data. Operational taxonomic unit (OTU) clustering and species classification were performed to acquire information about the species present and their abundance distribution.

### 2.5. Statistical Analysis

One-way analysis of variance (ANOVA) using SAS 8.2 for Windows (SAS Inc., Cary, NC, USA) was applied for the statistical tests. Differences between the means of each group were comparatively examined with the Duncan multiple comparison test and unpaired *t*-tests. The values were expressed as mean ± standard deviation. A value of *P* < 0.05 was considered the cutoff of statistical significance.

## 3. Results

### 3.1. Kaempferol Moderated Adipose Accumulation in HFD Mice

**Figure 1:**
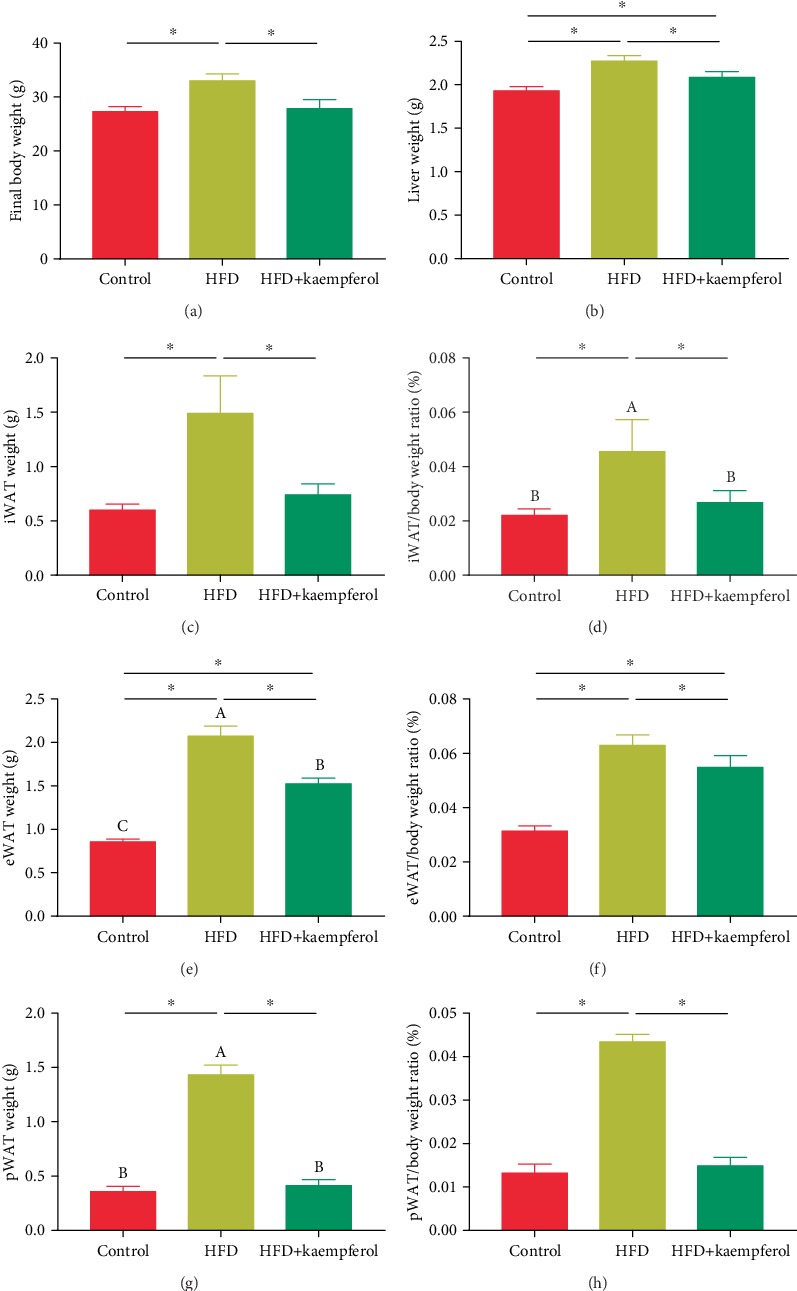
Body, liver, and adipose tissue weight measurements in the control, HFD, and HFD+kaempferol groups. The mice were randomly assigned to a control group, receiving a low-fat diet, an HFD group, receiving an HFD, and an HFD+kaempferol group, receiving an HFD and 200 mg/kg kaempferol (*n* = 10 each). (a) Body weight, (b) liver weight, (c) iWAT weight, (d) iWAT/body weight ratio, (e) eWAT weight, (f) eWAT/body weight ratio, (g) pWAT weight, and (h) pWAT/body weight ratio. Values are expressed as mean ± standard deviation. ^∗^*P* < 0.05.

Measurements of the body weight, liver, iWAT, eWAT, and pWAT were performed to assess the impact of kaempferol on HFD mice. As shown in Figures 1(a) and 1(b), the body and liver weight gain of HFD mice was higher than that of the control group (*P* < 0.05). However, it was lower in the HFD+kaempferol group than in the HFD group (*P* < 0.05). As shown in Figures 1(c)–1(h), the iWAT, eWAT, and pWAT were significantly heavier in the HFD mice compared to the control group (*P* < 0.05) but weighed less in the HFD+kaempferol group than in the HFD group (*P* < 0.05).

### 3.2. Kaempferol Ameliorated Hyperlipidemia in HFD Mice

**Figure 2:**
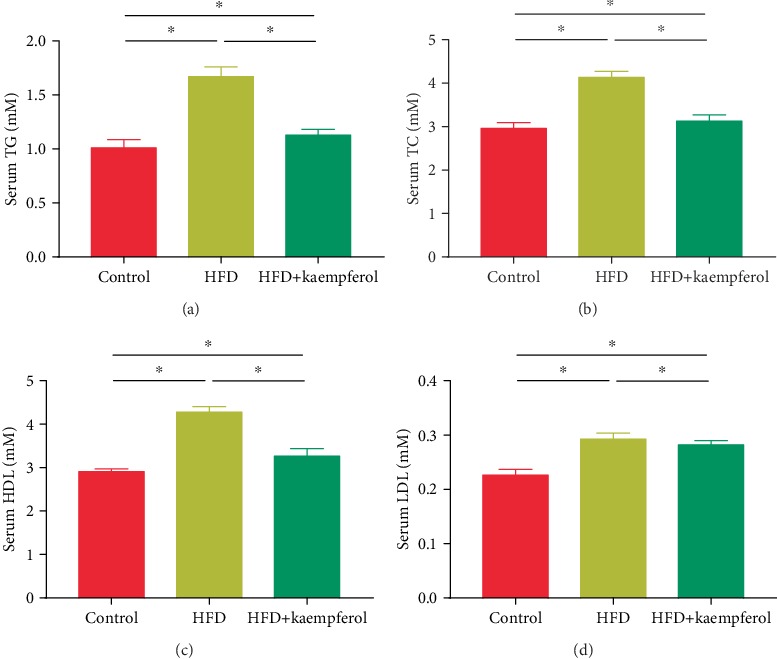
Serum concentrations of (a) total cholesterol (TC), (b) triglycerides (TG), (c) high-density lipoprotein (HDL), and (d) low-density lipoprotein (LDL) in the control, HFD, and HFD+kaempferol groups. Values are expressed as mean ± standard deviation (SD). ^∗^*P* < 0.05.

To examine the effect of kaempferol on the amelioration of hyperlipidemia, the concentrations of serum parameters were measured.  Figure 2 shows that serum TC, TG, HDL, and LDL concentrations were higher in the HFD mice compared to the control group (*P* < 0.05). However, kaempferol treatment led to significantly lower serum TC, TG, HDL, and LDL concentrations in the HFD+kaempferol group compared to the HFD group (*P* < 0.05).

### 3.3. Kaempferol Improved Glucose Tolerance and Insulin Resistance in HFD Mice

**Figure 3:**
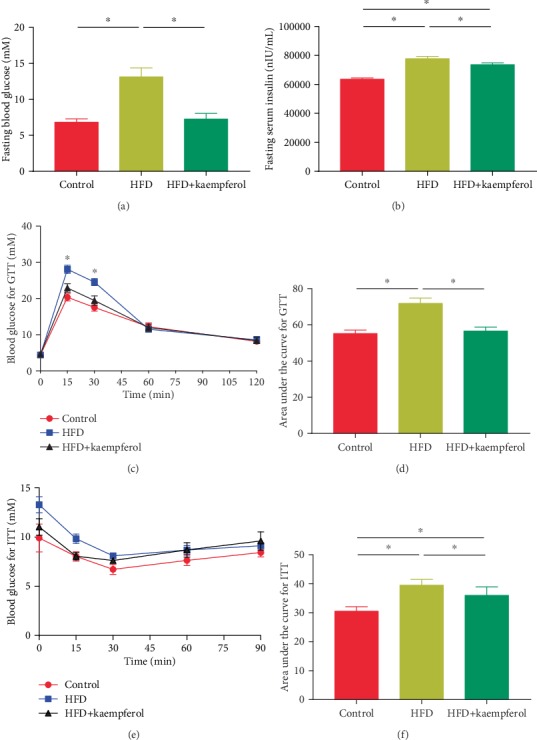
Serum concentrations of (a) blood glucose and (b) insulin, (c) curves of blood glucose levels, (d) calculated area under the curve for intravenous glucose tolerance tests (IGTT), (e) curves of blood glucose levels, and (f) calculated area under the curve for insulin tolerance tests in the control, HFD, and HFD+kaempferol groups. Values are expressed as mean ± standard deviation. ^∗^*P* < 0.05.

Given that obesity is linked to insulin resistance and glucose tolerance, an analysis of the concentrations of fasting blood glucose was performed.  Figure 3 shows that kaempferol supplementation kept fasting blood glucose to near-normal levels. Furthermore, intravenous glucose and insulin tolerance tests, paired with the corresponding values associated with the area under the curve, demonstrated that kaempferol led to less severe impairment of glucose tolerance and insulin resistance in the HFD+kaempferol group than in the HFD group.

### 3.4. Kaempferol Maintained Microbial Diversity and Altered Microbial Communities in HFD Mice

**Figure 4:**
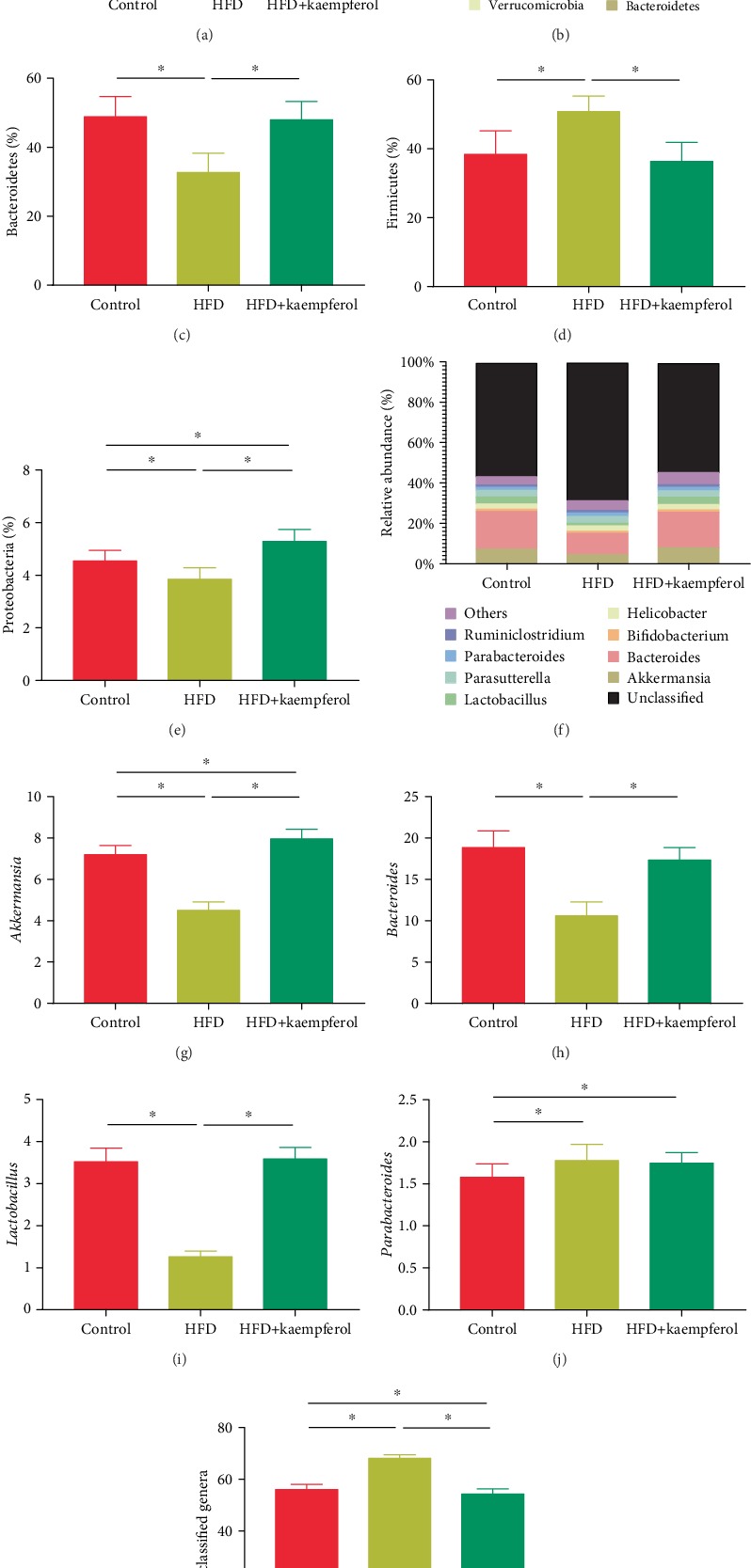
Gut microbiota analyses in the control, HFD, and HFD+kaempferol groups. (a) Shannon index in *α*-diversity analysis, (b) relative abundance of microbiota at the phylum level, (c) Bacteroidetes, (d) Firmicutes, and (e) Proteobacteria levels, (f) microbiota compositions at the genus level, (g) *Akkermansia*, (h) *Bacteroides*, (i) *Helicobacter*, (j) *Parabacteroides*, and (k) unclassified genus levels. Values are expressed as mean ± standard deviation. ^∗^*P* < 0.05.

The impact of kaempferol on gut microbiota compositions was assessed by sequencing the bacterial 16S rRNA V3-V4 region.  Figure 4 shows that gut microbiota diversity was lower in the HFD group than in the control group, as demonstrated by the higher Shannon indices in the latter (*P* < 0.05). Interestingly, the Shannon index in the HFD+kaempferol group was at the same level as that in the control group (*P* < 0.05). The total microbial compositions in the experimental groups at the phylum and genus levels are shown in Figures 4(b)–4(e) and 4(f)–4(k), respectively. The four main phyla in the three groups were Bacteroidetes, Firmicutes, Proteobacteria, and Verrucomicrobia (Figure 4(b)). Firmicutes were more abundant in the HFD group than in the control group (*P* < 0.05) and significantly less abundant in the HFD+kaempferol group than in the HFD group (*P* < 0.05). Conversely, Bacteroidetes and Proteobacteria were less abundant in the HFD group than in the control group (*P* < 0.05) and significantly more abundant in the HFD+kaempferol group than in the HFD group (*P* < 0.05). Moreover, Proteobacteria were significantly more abundant in the HFD+kaempferol group than in the control group (*P* < 0.05). Comparisons at the genus level revealed that *Akkermansia*, *Bacteroides*, and *Lactobacillus* were less abundant in the HFD group than in the control group (*P* < 0.05) and significantly more abundant in the HFD+kaempferol group than in the HFD group (*P* < 0.05). In addition, *Akkermansia* was more abundant in the HFD+kaempferol group than in the control group (*P* < 0.05). Unclassified genera were more abundant in the HFD group than in the control group (*P* < 0.05) and significantly less abundant in the HFD+kaempferol group than in the HFD group (*P* < 0.05).

## 4. Discussion

Pharmacological research indicates that kaempferol, a natural flavonoid found in various plant-based foods, is beneficial to human health. Antidiabetic activity is associated with kaempferol administration, including anti-inflammatory, antioxidative, and antihyperlipidemic effects, as well as protection of pancreatic *β*-cells [17]. However, findings regarding kaempferol's impact on gut microbiota and insulin resistance are scarce. Therefore, it is significant that in this study, kaempferol treatment was associated with fewer obesity-related complications in HFD mice. A statistically significant difference in body weight gain was observed between the HFD+kaempferol group and the HFD group. When treating obesity-related complications, reduction of fat storage and body weight is critical. Therefore, this study's results indicate that this natural flavonoid can be exploited as a cost-effective and safe compound for the treatment and prevention of HFD-induced obesity and insulin resistance.

In this study, kaempferol treatment led to differences in the epidermal white adipose tissue mass between the HFD+kaempferol group and the HFD group. Additionally, dietary kaempferol was associated with significantly less liver weight gain and enlargement compared to the HFD group. Moreover, in the HFD+kaempferol group, kaempferol kept body weight gain and elevation of blood glucose, serum cholesterol, and triglyceride levels lower than in the HFD group. It also resulted in lower hepatic lipid accumulation compared to the HFD group. One way to account for the increase in lipid metabolism associated with dietary kaempferol is by observing its impact on the regulation of hepatic expression of peroxisome proliferator-activated receptor *α* target genes [18]. Kaempferol may promote programmed cell death, thereby exerting an antitoxicological effect in various cancer cell types (e.g., pancreatic *β*-cells) [19]. Importantly, this effect is associated with concentrations of at least 30 *μ*mol/L, which is significant because dietary kaempferol levels are typically lower than 1 *μ*mol/L [20].

This study's data indicate that kaempferol administration improved insulin sensitivity and glucose tolerance in HFD mouse livers. *In vivo* experiments show that transcriptional regulation of lipogenesis genes (e.g., *Srebp-1c*, *Fas*, and *Scd*) is affected by insulin sensitivity changes in the liver [21]. Several *in vivo* studies indicate that obesity can be prevented by inhibiting hepatic lipogenesis and suppressing hepatic adipogenesis [22–24]. Thus, the preventive effect of kaempferol on obesity-related complications is possibly attributable to the weakened pathological processes of insulin resistance and inflammation [25].

The key to understanding obesity may be related to changes in the gut microbiota and its function in response to diet [26]. Several studies confirm that in both human and rat models, the gut microbiota in obesity exhibits greater relative abundance of Firmicutes and decreased levels of Bacteroidetes [27–29]. In line with these findings, this study found relative changes in the gut microbiota of HFD mice, showing that kaempferol supplementation led to lower levels of Firmicutes and higher levels of Bacteroidetes. However, the way in which kaempferol exerts these effects on the gut microbiota remains unclear. Its impact, particularly on Bacteroidetes, might be related to a consequent increase in the production of propionic acid, thereby mediating HFD-induced lipid metabolic dysfunction. These results are consistent with studies showing that an increase in propionate can attenuate weight gain in overweight human adults [30]. This mechanism may be linked to an increase in the secretion of gut hormones, including peptide YY and glucagon-like peptide-1 [30].

Overall, this study found that kaempferol administration in HFD mice moderated body and liver weight gain, hepatic lipid accumulation, and elevation of serum cholesterol, blood glucose, and triglyceride levels. The results also indicate that dietary kaempferol mediates the regulation of the gut microbiota, particularly by reversing the relative abundances of Firmicutes and Bacteroidetes to the levels observed in organisms with a healthy weight. This suggests that the gut microbiota may be implicated in the positive effect of kaempferol in HFD-induced lipid dysmetabolism. This study demonstrates that kaempferol supplementation has protective effects against insulin resistance and obesity in HFD mice, partly through mitigating insulin resistance deterioration and regulating the gut microbiota.

## Data Availability

All the data to support this study are available at the correspondence author upon request.

## References

[1] Ormazabal V., Nair S., Elfeky O., Aguayo C., Salomon C., Zuniga F. A. (2018). Association between insulin resistance and the development of cardiovascular disease. *Cardiovascular Diabetology*.

[2] Lee L., Sanders R. A. (2012). Metabolic syndrome. *Pediatrics in Review*.

[3] Zimmet P., Alberti K. G., Magliano D. J., Bennett P. H. (2016). Diabetes mellitus statistics on prevalence and mortality: facts and fallacies. *Nature Reviews Endocrinology*.

[4] Yang D., Hu C., Deng X. (2019). Therapeutic effect of chitooligosaccharide tablets on lipids in high-fat diets induced hyperlipidemic rats. *Molecules*.

[5] Ahechu P., Zozaya G., Martí P. (2018). NLRP3 inflammasome: a possible link between obesity-associated low-grade chronic inflammation and colorectal cancer development. *Frontiers in Immunology*.

[6] Jiang P., Ma D., Wang X. (2018). Astragaloside IV prevents obesity-associated hypertension by improving pro-inflammatory reaction and leptin resistance. *Molecules and Cells*.

[7] Wankhade U. D., Zhong Y., Kang P. (2018). Maternal high-fat diet programs offspring liver steatosis in a sexually dimorphic manner in association with changes in gut microbial ecology in mice. *Scientific Reports*.

[8] He C., Cheng D., Peng C., Li Y., Zhu Y., Lu N. (2018). High-Fat diet induces dysbiosis of gastric microbiota prior to gut microbiota in association with metabolic disorders in mice. *Frontiers in Microbiology*.

[9] Hussain A., Yadav M. K., Bose S. (2016). Daesiho-Tang is an effective herbal formulation in attenuation of obesity in mice through alteration of gene expression and modulation of intestinal microbiota. *PLoS One*.

[10] Kim J. Y., Cheon Y. H., Oh H. M. (2014). Oleanolic acid acetate inhibits osteoclast differentiation by downregulating PLC*γ*2–Ca^2 +^-NFATc1 signaling, and suppresses bone loss in mice. *Bone*.

[11] Ding S., Jiang H., Fang J. (2018). Regulation of immune function by polyphenols. *Journal of Immunology Research*.

[12] Zamora-Ros R., Knaze V., Romieu I. (2013). Impact of thearubigins on the estimation of total dietary flavonoids in the European Prospective Investigation into Cancer and Nutrition (EPIC) study. *European Journal of Clinical Nutrition*.

[13] Kumar Singh A., Cabral C., Kumar R. (2019). Beneficial effects of dietary polyphenols on gut microbiota and strategies to improve delivery efficiency. *Nutrients*.

[14] Murphy E. A., Velazquez K. T., Herbert K. M. (2015). Influence of high-fat diet on gut microbiota: a driving force for chronic disease risk. *Current Opinion in Clinical Nutrition and Metabolic Care*.

[15] Zheng G., Fan C., Di S. (2019). Ectopic expression of tea *MYB* genes alter spatial flavonoid accumulation in alfalfa (*Medicago sativa*). *PLoS One*.

[16] Alkhalidy H., Moore W., Wang A. (2018). Kaempferol ameliorates hyperglycemia through suppressing hepatic gluconeogenesis and enhancing hepatic insulin sensitivity in diet-induced obese mice. *The Journal of Nutritional Biochemistry*.

[17] Zhang Y., Liu D. (2011). Flavonol kaempferol improves chronic hyperglycemia-impaired pancreatic beta- cell viability and insulin secretory function. *European Journal of Pharmacology*.

[18] Chang C. J., Tzeng T. F., Liou S. S., Chang Y. S., Liu I. M. (2011). Kaempferol regulates the lipid-profile in high-fat diet-fed rats through an increase in hepatic PPAR*α* Levels. *Planta Medica*.

[19] Dang Q., Song W., Xu D. (2014). Kaempferol suppresses bladder cancer tumor growth by inhibiting cell proliferation and inducing apoptosis. *Molecular Carcinogenesis*.

[20] Barve A., Chen C., Hebbar V., Desiderio J., Saw C. L.-L., Kong A.-N. (2009). Metabolism, oral bioavailability and pharmacokinetics of chemopreventive kaempferol in rats. *Biopharmaceutics & Drug Disposition*.

[21] Sanders F. W. B., Griffin J. L. (2016). *De novo* lipogenesis in the liver in health and disease: more than just a shunting yard for glucose. *Biological Reviews of the Cambridge Philosophical Society*.

[22] Xing S. F., Liu L. H., Zu M. L. (2018). The inhibitory effect of gypenoside stereoisomers, gypenoside L and gypenoside LI, isolated from *Gynostemma pentaphyllum* on the growth of human lung cancer A549 cells. *Journal of Ethnopharmacology*.

[23] Kao E. S., Yang M. Y., Hung C. H., Huang C. N., Wang C. J. (2016). Polyphenolic extract from *Hibiscus sabdariffa* reduces body fat by inhibiting hepatic lipogenesis and preadipocyte adipogenesis. *Food & Function*.

[24] Liu H., Liu M., Jin Z. (2019). Ginsenoside Rg2 inhibits adipogenesis in 3T3-L1 preadipocytes and suppresses obesity in high-fat-diet-induced obese mice through the AMPK pathway. *Food & Function*.

[25] Luo C., Yang H., Tang C. (2015). Kaempferol alleviates insulin resistance via hepatic IKK/NF-*κ*B signal in type 2 diabetic rats. *International Immunopharmacology*.

[26] Federico A., Dallio M., di Sarno R., Giorgio V., Miele L. (2017). Gut microbiota, obesity and metabolic disorders. *Minerva Gastroenterologica e Dietologica*.

[27] Duan Y., Zhong Y., Xiao H. (2019). Gut microbiota mediates the protective effects of dietary *β*‐hydroxy‐*β*‐methylbutyrate (HMB) against obesity induced by high-fat diets. *The FASEB Journal*.

[28] Chang C. J., Lin C. S., Lu C. C. (2017). Correction: Corrigendum: *Ganoderma lucidum* reduces obesity in mice by modulating the composition of the gut microbiota. *Nature Communications*.

[29] Chang C. J., Lin C. S., Lu C. C. (2015). *Ganoderma lucidum* reduces obesity in mice by modulating the composition of the gut microbiota. *Nature Communications*.

[30] Chambers E. S., Viardot A., Psichas A. (2015). Effects of targeted delivery of propionate to the human colon on appetite regulation, body weight maintenance and adiposity in overweight adults. *Gut*.

